# Patent vitellointestinal duct with ileal prolapse in a newborn: A case report and literature review

**DOI:** 10.1097/MD.0000000000036919

**Published:** 2024-01-19

**Authors:** Feng Chen, Yong Zeng, Lin-Lin Yue, Ming-Feng Xie, Hai-Jin Liu

**Affiliations:** a Department of Pediatric Surgery, The First Affiliated Hospital of Gannan Medical University, Ganzhou, China; b Jiangxi Provincial Clinical Research Center for Vascular Anomalies, The First Affiliated Hospital of GanNan Medical University, Ganzhou, China; c Department of Intensive Care Unit, The First Affiliated Hospital of Gannan Medical University, Ganzhou, China.

**Keywords:** intestinal prolapse, newborn, patent vitellointestinal duct

## Abstract

**Rationale:**

Patent vitellointestinal duct is the most common omphalomesenteric duct anomaly to present with symptoms.

**Patient concerns:**

A 10-day-old child presented with increase in the size of a polypoidal lesion into a large, “Y”-shaped reddish, prolapsing lesion, discharging gaseous, and fecal matter at her umbilicus. A laparoscopic exploration was performed, followed by wedge resection and anastomosis. No complications occurred during postoperative follow-up.

**Diagnoses:**

A patent vitellointestinal duct with ileal prolapse.

**Interventions:**

The resection of extended intraperitoneal intestinal tube was performed.

**Outcomes:**

During the follow-up 3 months after surgery, the umbilical cord of the child healed well after surgery.

**Lessons:**

Timely surgical treatment can minimize the occurrence of complications, and the overall prognosis is good after surgery.

## 1. Introduction

The vitellointestinal tube is a special structure in the fetus that connects the midgut to the vitellointestinal sac and provides nutrients to the fetus until the placenta is formed. It usually gradually subsides at 5 to 9 weeks of gestation, and abnormal degeneration of the vitellointestinal duct will lead to a series of congenital malformations.^[1]^ Among them, Meckel diverticulum is the most common anomalies. Patent vitellointestinal duct is one of the rarest abnormalities of vitellointestinal duct degeneration, with an incidence of about 0.0053%, meanwhile ileal prolapse in neonates is even rarer.^[1,2]^ We present a rare case of an ileal prolapse through patent vitellointestinal duct in a 10-day-old child. Through literature review, we have summarized the diagnosis and treatment of neonatal umbilical bowel fistula complicated with ileal prolapse.

## 2. Case presentation

**Figure 1.**
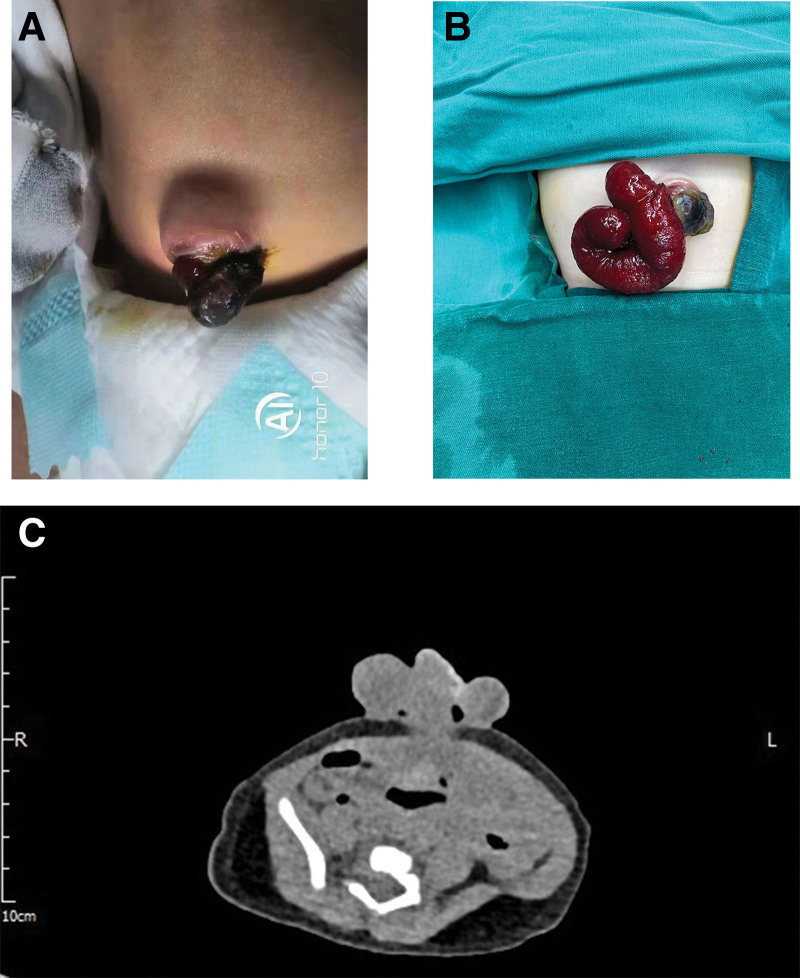
Umbilical lesions. (A) Red mass in the umbilical cord; (B) the red mass protruding from the umbilical cord gradually increased in the shape of “Y”; (C) CT imaging examination of the lesion.

The patient was a 10-day-old female, full-term infant, who was admitted to hospital 3 days after the discovery of a red mass in the umbilical cord. She had no special personal history or maternal history. After birth, the child was found to have abnormal umbilical bulge, accompanied by sticky secretions and no overflow of feces (Fig. 1A). Then she was admitted to a local hospital and treated with anti-infection and umbilical cord nursing. During the hospitalization, the child developed a red mass protruding from the umbilical cord. After violent crying, the red mass protruding from the umbilical cord gradually increased in the shape of “Y” (Fig. 1B). After the lesion was wrapped in sterile saline gauze, the patient was transferred to the emergency department of our hospital.

The physical examination showed that the abdomen was slightly distended, the abdominal muscle was soft, the umbilical cord stump was not removed. A “Y” shaped intestinal tubular red mass was prominent at the left edge of the umbilical cord root, and there was a similar fistula at 2 stump points. No leakage of feces, negative mobility flap, and normal bowel sound were found in this child. Abdominal CT examination showed that the umbilical cord was enlarged and irregular soft tissue was protruding. There were multiple intestinal shadows within the umbilical cord and the range was about 48 mm × 24 mm. Some intestinal dilatation and gas accumulation in the abdominal cavity were also found (Fig. 1C).

## 3. Treatment

The child had umbilical cord protrusion and significant intestinal redness and swelling, and there was a risk of intestinal strangulation. Emergency operation was performed after admission. First, laparoscopic exploration showed that the ileum was herniated into the umbilical cord in a “folding shape,” which continued with the protruding intestinal tube in the umbilical cord, and the color of the intestinal tube in the abdominal cavity was normal. After the pneumoperitoneum was closed, a circular incision was made along the umbilicus, the skin and subcutaneous tissue were resected, the umbilical vein and umbilical artery were separated and sutured, and the extended intraperitoneal intestinal tube was pulled out from the umbilical incision. It was confirmed that the proximal and distal transumbilical intestinal fistula of the ileum presented bidirectional prolapse in vitro. The intraoperative diagnosis was neonatal umbilical intestinal fistula with bidirectional ileal prolapse. After the resection, no ischemic necrosis was found in the bowel. It was found that the fistula was connected to the ileocecal tube in a “T” shape. The fistula was about 1.5 cm long, 0.6 cm in diameter, and 60 cm away from the ileocecal part. An intestinal forceps was placed on each side of the fistula and intestinal duct transition, and the fistula was gently lifted. A cutting and closing device was used to remove the fistula completely and close the intestinal duct. After closure, it was checked that the intestinal duct was unobstructed without stenosis, blood leakage, and gas leakage. The intestinal tube was placed into the abdominal cavity in order, and then the umbilical fossa was reconstructed.

## 4. Follow-up

**Figure 2.**
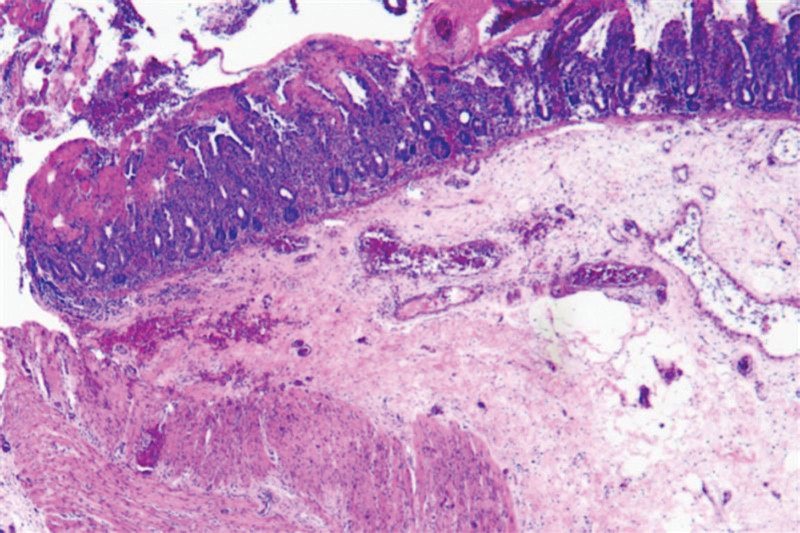
Postoperative pathology of the lesion.

**Figure 3.**
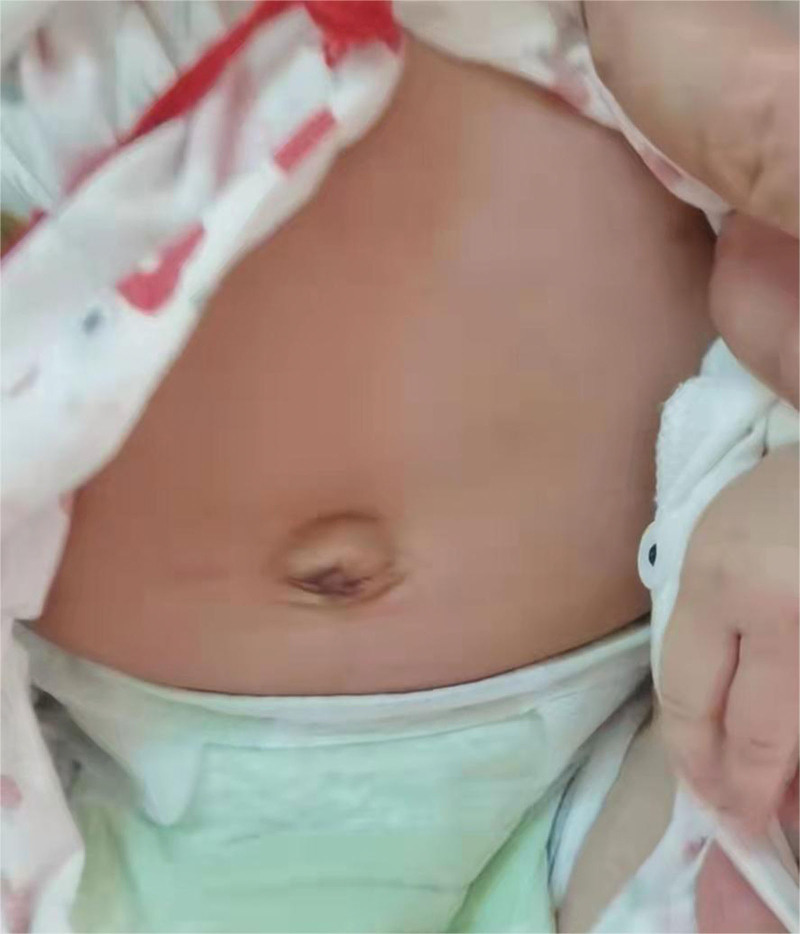
Follow-up umbilical appearance at 3 months after surgery.

The patient was transferred from the neonatal intensive care unit to the general ward 2 days after surgery. The postoperative pathological results showed that the intestinal mucosa was observed under microscope, focal mucosal glands were necrotic and exfoliated, interstitial blood vessels were dilated and hyperemia, and there was a large amount of lymphocytoplasmic cell infiltration, which was consistent with the changes of umbilical intestinal fistula (Fig. 2). Three days after the operation, the child had defecated and vented by herself, and was breastfed. Postoperative review of intestinal ultrasound showed no intestinal fistula, intestinal obstruction or other abnormalities. This child was discharged successfully on the 7th day after the operation. During the follow-up 3 months after surgery, the umbilical cord of the child healed well after surgery, with no abdominal distension or intestinal obstruction (Fig. 3).

## 5. Literature review

The literature was published from June 1992 to June 2022 in Chinese Wanfang Database, PubMed, Google Scholar, Springer were searched using key words “patent vitellointestinal duct” and “intestinal prolapse.” The inclusion criteria were suitable for umbilical intestinal fistula complicated with intestinal prolapse. The reports with incomplete clinical information were excluded.

Fifteen relevant literatures were found and 9 reports (11 cases in total) were summarized after excluding the reports with incomplete data. All the children underwent surgical treatment, 8 of them underwent umbilical annular incision, and 2 of them underwent subumbilical transverse incision or umbilical spindle incision due to intestinal rupture or gangrene. Fistula and partial intestinal resection and end to end intestinal anastomosis were performed in 9 cases, and fistula wedge resection and transverse intestinal anastomosis were performed in 2 cases. There were 2 cases of ileum necrosis and 1 case of ileum rupture. There were no deaths and no obvious complications during postoperative follow-up. The clinical data of 11 cases are summarized in Table 1.

**Table 1:** Literature review of the 11 cases.

Literature	Age of case	Surgical protocol	Prognosis
Mohite PN et al^[3]^	5 months	A 4 cm transverse incision below the umbilical cord, fistula resection + ileostomy	Follow-up for 6 months without complications
Patel RV et al^[2]^	10 days	Cord ring incision, fistula resection + ileal end anastomosis	Follow-up for 3 months without complications
Singh S et al^[4]^	2 months	Cord ring incision, fistula resection + ileal end anastomosis	Follow-up for 6 months without complications
Singh S et al^[4]^	1.5 month	Cord ring incision, fistula resection + ileal end anastomosis	-
Borkar N et al^[5]^	1 day	Cord ring incision, fistula resection + ileal end anastomosis	–
Tadesse A et al^[6]^	4 days	Cord ring incision, fistula resection + gangrenous bowel resection + end anastomosis	–
Handayani H et al^[7]^	7 days	A midline longitudinal incision was made with fistula resection + ruptured ileectomy + ileal terminal anastomosis	Follow-up for 3 weeks without complications
Basus class^[8]^	1 month	The umbilical incision was extended, fistula resection + gangrenous ileectomy + ileal terminal anastomosis	–
Dong Shuai Jun et al^[9]^	22 days	Cord ring incision, fistula resection + ileum rampant anastomosis	–
Xu Bing et al^[10]^	2 days	Subumbilical arc incision, fistula resection + ileum rampant anastomosis	Follow-up for 6 months without complications
Xu Bing et al^[10]^	13 days	Cord spindle incision, fistula resection + gangrenous bowel resection + end anastomosis	Follow-up for 6 months without complications

## 6. Discussion

Umbilical intestinal fistula can be caused by complete patent of the vitellointestinal duct. When the fistula is thick or the distal intestinal duct is obstructed, the intestinal canal is prone to prolapse through the fistula through the umbilical cord, and the mucosa is everted to form intussusception and incarceration similar with that from the umbilical cord. One pore can be seen at the top of the intestinal prolapse, and 2 pores can be found in the 2-way prolapse. When the intestinal canal is slightly prolapsed, the umbilical bulge is columnlike, and there is no typical “fecal overflow” of the umbilical intestinal fistula, only intestinal fluid or other secretions. If the intra-abdominal pressure of the child continues to increase, the proximal and distal intestinal tubes of the fistula will turn out at the same time, showing a typical “Y” shape.^[10]^

**Figure 4.**
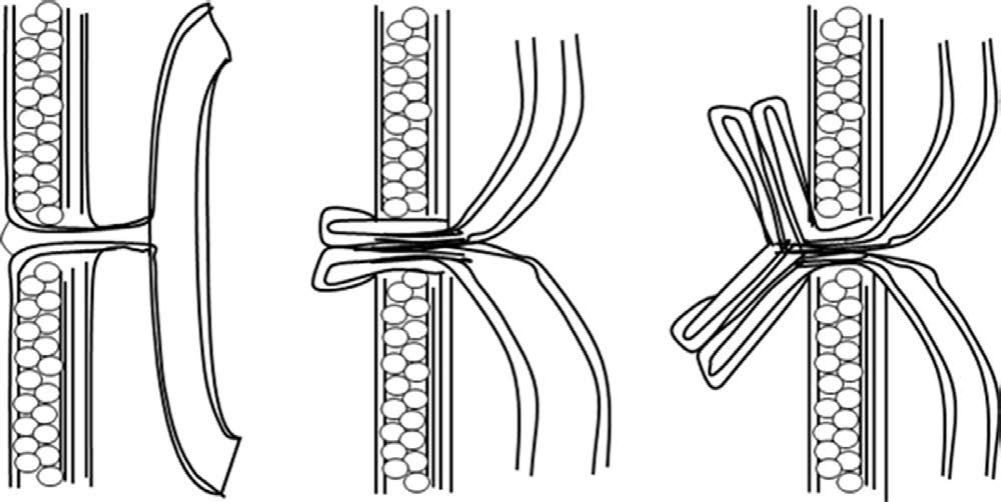
Schematic diagram of disease progression of patent vitellointestinal duct with ileal prolapse.

Patent vitellointestinal duct complicated with ileal prolapse is rare. Howard S et al firstly reported this disease in the Lancet in 1953,^[11]^ so far only a few cases have been reported. Based on the clinical observation of this child, we drew a schematic diagram of neonatal umbilical bowel fistula complicated with ileal prolapse to facilitate the understanding of the progression of this disease and help clinicians to improve the ability of early diagnosis of this disease (Fig. 4).

Early diagnosis of patent vitellointestinal duct complicated with ileal prolapse can prevent serious complications such as intestinal ischemic necrosis. Prenatal ultrasonography has certain advantages in the diagnosis. The umbilical herniation can be seen and does not disappear in continuous ultrasonography. However, if it is completely covered by the umbilical cord or the umbilical cord is not obvious, it is difficult to diagnose and can easily be misdiagnosed as small omphalocele.^[12]^ After birth, the red tubular structure of intestinal mucosa with eversion should be highly suspected of umbilical fistula complicated with ileal prolapse. When the fistula and intestinal canal are slightly prolapsed and the umbilical bulge is columnar, it should be distinguished from small umbilical bulge rupture and small intestinal entrapment. Small omphalocele rupture and small intestinal incarceration can also manifest as hyperemia, red and swollen bowel protrusion, but there is no mucosal ectropion in the puffed bowel, and the bowel is in continuous shape without blind end. Abdominal CT plain scan and gastrointestinal angiography can be performed to further identify the disease.

Neonates with patent vitellointestinal duct complicated with ileal prolapse should be treated with timely surgery. Delay of operation may lead to the progression of intestinal prolapse, strangulation intestinal obstruction, intestinal necrosis, or even death.

If the diagnosis is not clear, we suggest that laparoscopic exploration can be performed firstly. Annular incision of the umbilical cord can be performed, and the original umbilical ring can be retained as far as possible. If the base of the umbilical bowel fistula is narrow and there is no obvious abnormality of the bowel condition, wedge resection of the umbilical bowel fistula and intestinal transverse suture can be performed. If the base is wide or accompanied by intestinal ischemic necrosis or intestinal strangulation, umbilical intestinal fistula, resection, and anastomosis are recommended.

According to the literature review, the prognosis of patent vitellointestinal duct complicated with ileal prolapse is better after active surgical treatment without serious complications. In this case, also no complications occurred during postoperative follow-up.

There were also some limitations in this study. First, this is a case report. Further study with large sample size is needed. Second, there is only 3 months follow up for this case. Longer follow up is needed.

To sum up, the typical symptoms of neonatal umbilical bowel fistula complicated by ileal prolapses are the gradual enlargement of the red mass protruding from the umbilical cord, which is in the shape of a column or “Y” shape, with visible pores at the top. Due to the rare occurrence of umbilical bowel fistula complicated with ileal prolapse in neonates, there is a certain lack of understanding, which leads to delayed treatment and even the wrong treatment plan. After the discovery, timely surgical treatment can minimize the occurrence of complications, and the overall prognosis is good after surgery.

## Author contributions

**Conceptualization:** Feng Chen, Yong Zeng, Lin-Lin Yue, Ming-Feng Xie, Hai-Jin Liu.

**Data curation:** Feng Chen, Yong Zeng, Lin-Lin Yue, Ming-Feng Xie, Hai-Jin Liu.

**Investigation:** Feng Chen, Yong Zeng, Lin-Lin Yue, Ming-Feng Xie, Hai-Jin Liu.

**Methodology:** Feng Chen, Yong Zeng, Lin-Lin Yue, Ming-Feng Xie, Hai-Jin Liu.

**Writing – original draft:** Feng Chen, Yong Zeng, Lin-Lin Yue, Ming-Feng Xie, Hai-Jin Liu.

**Writing – review & editing:** Feng Chen, Yong Zeng, Lin-Lin Yue, Ming-Feng Xie, Hai-Jin Liu.
